# Adhesive Joints of Additively Manufactured Adherends: Ultrasonic Evaluation of Adhesion Strength

**DOI:** 10.3390/ma15093290

**Published:** 2022-05-04

**Authors:** Jakub Kowalczyk, Dariusz Ulbrich, Kamil Sędłak, Michał Nowak

**Affiliations:** 1 Faculty of Transport and Civil Engineering, Institute of Machines and Motor Vehicles, Poznan University of Technology, 60-965 Poznań, Poland; dariusz.ulbrich@put.poznan.pl; 2 Faculty of Mechanical Engineering, Division of Virtual Engineering, Poznan University of Technology, 60-965 Poznań, Poland; kamil.sedlak@gmail.com (K.S.); michal.nowak@put.poznan.pl (M.N.)

**Keywords:** adhesion, additive technology, aluminum, structural adhesive, ultrasound, damping, strength

## Abstract

Adhesive joints are widely used in the construction of machines and motor vehicles. Manufacturers replace them with the welding and spot-welding methods due to the lack of damage to the material structure in the joint area. Moreover, it is aimed at reducing the weight of vehicles and producing elements with complex shapes. Therefore, additive manufacturing technology has been increasingly used in the production stage. This fact has not only changed the view on the possibilities of further development of the production technology itself, but it has also caused an intense interest among a greater number of companies in the advantages of structural optimization. There is a natural relationship between these two areas in the design and production, allowing for almost unlimited possibilities of designing new products. The main goal of the research described in this article was to determine the correlation between the strength of the adhesive joint of elements produced using additive technology and the parameters of the ultrasonic wave propagating in the area of the adhesive bond. The tests were carried out on samples made of AlSiMg0.6 material and a structural adhesive. Strength tests were performed to determine the shear force which damaged the joint. Furthermore, an ultrasonic echo technique enabling the determination of a nondestructive measure of the quality and strength of the joint was developed. The samples of the adhesive joints had a strength of about 18.75–28.95 MPa, which corresponded to an ultrasonic measure range of 4.6–7.8 dB. The determined regression relationship had a coefficient of determination at the level of 0.94. Additional ultrasonic tests of materials made with the additive technology confirmed its different acoustic properties in relation to aluminum produced with the standard casting or extrusion process. Designated dependence combining the mechanical strength and the decibel difference between the first and second impulses from the bottom of the joint may constitute the basis for the development of a nondestructive technique for testing the strength of adhesive joints.

## 1. Introduction

Manufacturers of machines and vehicles aim to reduce weight, which leads to a reduction of fuel consumption and the emission of fumes into the atmosphere [1,2,3]. The ecological aspect of the design process of machines and devices plays a significant role in the selection of materials and connections used at the production stage. One of the directions of development of the production process of machine elements is the use of additive manufacturing technology (3D printing) in metals and their alloys [4,5,6]. Thanks to this technology and the use of shape optimization [7,8], parts with a reduced mass but a similar or higher strength compared to currently manufactured elements are obtained. Moreover, efforts are being made to improve the technology of joining individual parts by various methods, including spot welding [9,10], welding [11], as well as bonding with adhesives [12,13]. In addition, various types of coatings are used at the production stage, which change the properties of the surface layer [14,15].

The development of devices used for the production of parts using additive manufacturing technology makes it necessary to test various properties of the manufactured products [16,17,18]. Additive technology is a process controlled by a computer in which the three-dimensional object is produced by depositing multiple layers of equal structure. The individual layers of the material with the same thickness are placed on top of each other (layer by layer), which leads to obtaining objects of the desired shapes and dimensions without additional processing. The thickness of the applied layers may vary from 10 to 200 µm depending on the equipment used and the process parameters. Each subsequent layer in the construction of an object is added as a result of the melting or partially melting of the material. Other processes such as the sintering or polymerization of materials in predetermined layers with no needs of tools have been used. Typically, powders of metals or other materials are used, depending on the properties of the final product. In the powder system, the deposited powder is spread using a roller or a squeegee; in some systems the material is applied through a nozzle that deposits the required material. The main directions of research on elements manufactured in additive manufacturing include the improvement of manufacturing technology [19,20,21], identification of defects [22,23], including mainly the lack of homogeneity of the material, which causes different material properties and may lead to the detachment of individual layers of the structure [24]. Another research is focused on the optimal performance of elements manufactured using additive technology [25]. An incorrect design of the manufacturing process, e.g., support bugs, may cause both shape errors of the element as well as the consumption of an additional, large amount of powder, which generates significant costs (supports are removed and treated as material waste). Nevertheless, there is a gap in the study of the acoustic properties of aluminum-based additive components and the comparison of these properties with the results obtained for aluminum samples produced in standard manufacturing processes (e.g., casting, extrusion). Supplementing the knowledge in this area can provide the basis for the development of an ultrasonic quality assessment method for both the elements made in additive technology as well as the inseparable connections which these elements contain.

One of the methods of joining elements manufactured in additive technology is bonding with an adhesive. Adhesive joints are classified as inseparable joints and are commonly used in the construction of motor vehicles and machines. The main directions of research on adhesive joints include, above all, the design of joints of various materials [26], which have the properties required for a given joint, testing the strength of adhesive joints of various materials exposed to a complex state of stress [27,28], as well as the development of nondestructive testing techniques which allow for the location of defects in the connection [29,30]. The use of the ultrasonic method to test adhesive joints is described in the literature [31,32]. Nevertheless, the performed tests include tests of adhesive joints made of standard materials (rolled sheets, forgings, castings, carbon fiber), the acoustic properties of which have been known for many years [33]. In the case of examining the adhesive joints of elements made with the use of additive technology, changes in the internal structure of the material are observed [34], which may affect the propagation of the ultrasonic wave and the obtained information on the condition of the connection. Ultrasonic tests of adhesive joints include the location of defects and continuity of the adhesive path [35,36], kissing-bond verification [37] and, in a few attempts, the assessment of the joint production process [38]. According to the authors, there is one more important research direction related to the development of a nondestructive method of estimating the strength of adhesive joints. This applies to both standard connections and those made of metal in additive technology.

The main aim of the research presented in this article was to determine the relationship between the mechanical strength of the adhesive joint of two flat bars made using additive technology, and the parameters of the ultrasonic wave propagating in the area of this joint. An additional goal was to determine the basic acoustic properties of a sample made of AlSiMg0.6 material (produced with the use of additive manufacturing technology) and to compare it with standard aluminum (which was produced using traditional methods). The research includes the selection of the shape of the samples and adhesives for preparing the adhesive joint, ultrasonic equipment choice, as well as the determination of the selected ultrasonic measure of the joint quality and shear strength. The final result is a mathematical dependence linking the strength of the adhesive bond with the selected ultrasonic measure. This is the first step in the development of a method and system for estimating the strength of adhesive joints using ultrasonic waves, both classic and made using additive manufacturing technology.

## 2. Materials and Methods

### 2.1. Samples

The samples of AlSiMg0.6 powder were manufactured and then melted with a laser beam (Table 1). The samples were manufactured with the LPBF/SLM process using “A357—As-built” powder. The samples were made in such a way as not to affect the internal structure of the material. The surface finish–raw surface was treated as described in the sample preparation process. Only vertical specimens were left for inclination of the specimen relative to the work platform during fabrication. Therefore, the surface with the best properties was obtained. The manufacturing process of the specimens was not influenced by other factors related to the process, parameters, size of the workpiece, presence of supporting structures and residues of structures. This process was influenced by the surface roughness and waviness. Material properties-A357 were as follows: tensile strength 380 MPa, yield strength 250 MPa, elongation 8%, fatigue strength 80 MPa, Young’s modulus ~70 GPa.

All samples investigated in this research were manufactured using the LPBF process, from AlSi7Mg0.6 powder of particle distribution 20–63 μm (SLM Solutions AG, Lubeck, Germany) using the process parameters which allowed us to obtain almost fully dense material (porosity < 0.5%). The samples during the build with the LPBF process were oriented vertically—the longitudinal axis was aligned in the build direction along the z axis without any inclination. This orientation was optimal because of the minimal required supporting area (reduced only to the smallest sample edge), and due the fact that the surface quality on the side surfaces of the specimens was uniform and had the smallest achievable roughness. Additionally, a vertical orientation minimized residual stresses, which were introduced into the material during the processing in AM.

**Figure 1:**
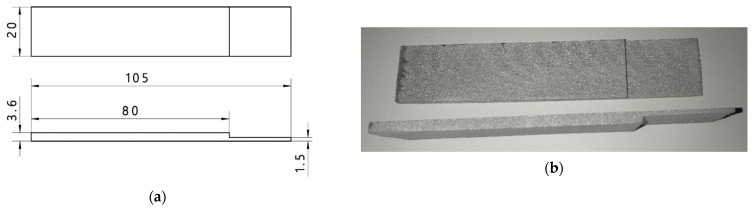
Samples used during the test; (**a**) scheme with dimension, (**b**) view of the sample.

The thickness of the sample at the place where the adhesive joint was made was 1.5 mm, which corresponds to the thickness of the elements currently used in the construction of car bodies of motor vehicles. Additionally, such a thickness of the sample prevents its damage (due to the action of shear forces, the adhesive joint, rather than the sample material, is damaged). The length of the sample was set at 105 mm, which allowed it to be easily mounted on a testing machine. In addition, the reduction in material thickness at the point of bonding the samples was due to the need to maintain the alignment (axially) of the samples during the shear test. In the case of samples of the same thickness, the tearing and deformation of the sample material occur apart from the action of shear forces. This proves the occurrence of a complex state of stress. Both in ultrasonic tests and in the shear test, 20 sets of samples made with the additive technology were used. The dimension and view of one sample is presented in Figure 1.

**Figure 2:**
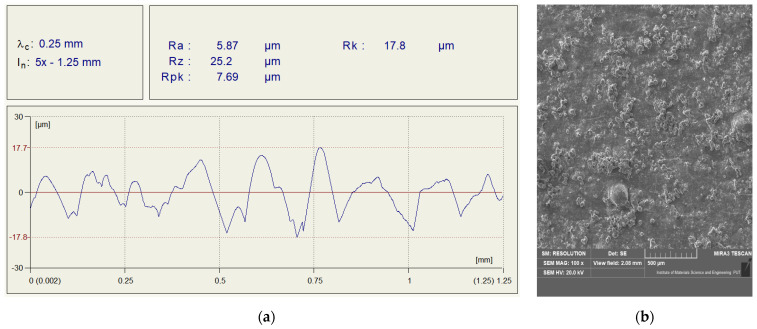

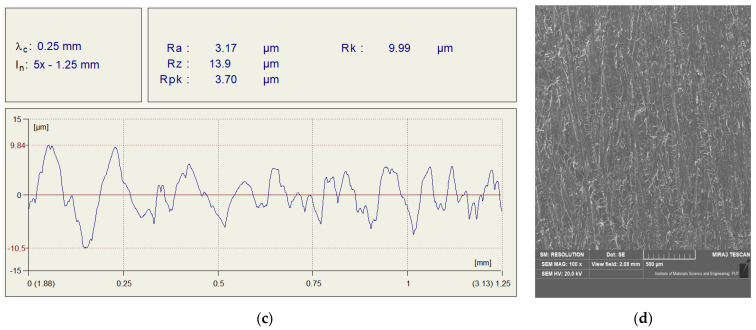
Assessment of the surface layer before the bonding process; (**a**) surface roughness profile after sandblasting, (**b**) view of the surface structure after sandblasting, (**c**) surface roughness profile after sanding with P80 abrasive paper, (**d**) view of the surface structure after sanding with P80 abrasive paper.

After the additive manufacturing process, the surface required additional processing. Therefore, 3 methods were used to prepare the surface layer of the samples before bonding. The first one consisted of degreasing (an isopropanol-based degreaser was used). The second method of surface preparation was abrasive blasting (sandblasting). However, the last method of surface preparation was the use of a P80 abrasive paper, which corresponded to the appropriate grain diameter. Examples of the measurement results of the surface roughness profile and the surface view observed on an SEM microscope after treatment are shown in Figure 2. In the case of all tested sanded samples, the obtained values of the R_a_ parameter fell within the range of 5.75–5.94. On the other hand, the second parameter R_z_—important from the point of view of the bonding process—reached values in the range of 24.5–25.8. For the second sample surface preparation method (grinding with a P80 abrasive paper), values of R_a_ and R_z_ within the range of 2.91–3.45 and 12.9–14.1 were obtained, respectively. In the case of the R_a_ parameter for both methods of surface preparation, the results were at a similar level.

Four adhesives with different components and properties were used to connect the samples, in order to select one adhesive which will be used in the further part of the research (main research). The course of the bonding process was always in line with the technological card of the adhesive manufacturer. The most important parameters of adhesives used in the tests are summarized in Table 2. Setting time is the time to obtain the handling strength. Cure time is the time to obtain the full mechanical strength, All the adhesives were cured at room temperature (around 20 °C).

### 2.2. Ultrasonic Testing of Samples and Joints

Ultrasonic testing was divided into two stages. In the first of them, the acoustic properties of the samples produced with the additive manufacturing technology were measured. The second stage involved the ultrasonic testing of adhesive joints with the use of the selected adhesive (adhesive 4—previously used for bonding samples).

Ultrasonic testing uses mechanical waves that propagate through the material and cause vibrations of the material particles. Ultrasound is based on the theory of acoustoelasticity, and the mechanical stresses in the material have an impact, for example, on the propagation speed and other properties of the ultrasonic wave. The velocity of the longitudinal wave propagation can be determined on the basis of the relationship, taking into account the impulses from the bottom of the sample at the same material thickness. Longitudinal wave velocity can be related to other parameters characterizing the tested material, such as, Young’s modulus, Poisson’s ratio or material density:

(1) $$V_{L}=\sqrt{\frac{E\left(1-v\right)}{\rho \left(1-v\right)\left(1-2v\right)}}$$

Other factors influencing the results of ultrasonic tests are surface roughness (at the point of wave penetration), anisotropy and micro-heterogeneity of the material. The microstructure of the material, including the size of the grain produced in the production process (i.e., additive manufacturing technology), is the factor that determines the attenuation of ultrasonic wave. The attenuation of the ultrasonic wave in the material occurs due to the scattering and absorption of waves. The result of the damping of the ultrasonic wave is the amount of energy that returns to the ultrasonic head and is displayed in the form of an A-scan (amplitude vs. time signals) on the ultrasonic flaw detector (GE Sensing & Inspection Technologies, Billerica, MA, United States) screen.

**Figure 3:**
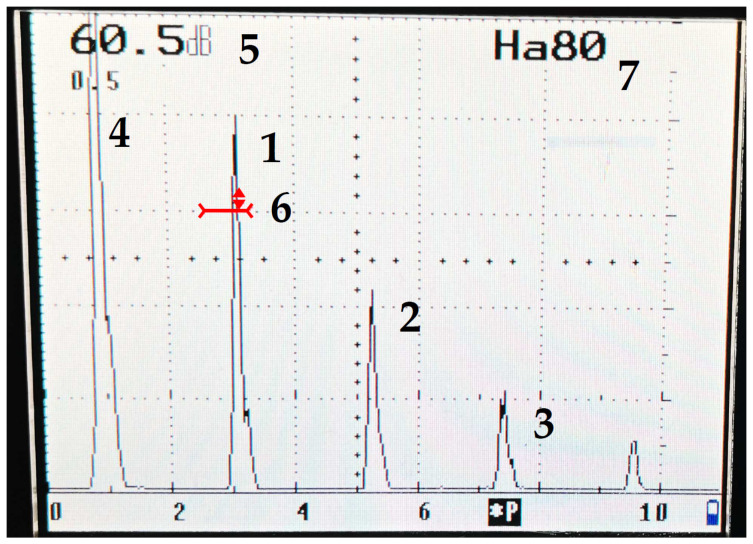
Ultrasonic flaw detector; 1—first pulse (H_I_), 2—second pulse (H_II_), 3—third pulse (H_III_), 4 —transmit impulse, 5—gain, 6 —gate (red color), 7—amplitude value of gate pulse.

Our research started with determining the damping of the ultrasonic longitudinal wave propagating in the sample made using the additive technology with AlSi7Mg0.6. In order to determine the attenuation, the amplitude (height) of the first five pulses (Figure 3) obtained on the screen of the ultrasonic flaw detector was measured 30 times. Measurements were made on samples with a thickness of 3.6 mm. Before testing, the surface of the element was cleaned and degreased. No surface treatment which could affect the propagation of the ultrasonic wave was performed.

The GE 3.15 G20MNX ultrasonic head (GE Sensing & Inspection Technologies, Billerica, MA, United States) was selected for the tests. This head allows one to generate a system of impulses on the flaw detector screen in an element of small thickness. It is a head with a transducer frequency of 20 MHz and a diameter of 3.15 mm with a water delay line, enabling measurements outside the dead zone. It is important from the point of view of the research accuracy and obtained results.

The attenuation coefficient was determined using relationship (2). It was decided that only the first two pulses would be selected to calculate the damping because the measurement error for these pulses was the lowest.

(2) $$\propto_{f}=\frac{20}{2l}\cdot \log\left(\frac{H_{I}}{H_{\mathrm{II}}}\right)$$

where: H_I_, H_II_—percentage height (the amplitude value) of the first and second impulse from the bottom of the tested element, respectively, l—thickness of the element.

Ultrasonic wave attenuation is defined as the conversion of the propagation energy of an ultrasonic wave of a specified frequency into vibration energy at other frequencies, usually thermal vibrations. This transformation is influenced by physical mechanisms, such as: thermoelastic damping, damping due to structural relaxation phenomena (it has a similar course to resonance as a function of frequency), resonance damping and damping caused by dislocation vibrations as well as damping caused by detaching dislocations from contaminants (depending on the vibration amplitude). It is important that for a relaxation process in which the same activation energy is for all systems subject to relaxation, the shape of the curve of the damping coefficient $Q^{-1}$(f) as a function of frequency is described by the expression (3):

(3) $$Q^{-1}\left(f\right)={\left(Q^{-1}\right)}_{\max}\frac{2\frac{f}{f_{\max}}}{1+{\left(\frac{f}{f_{\max}}\right)}^{2}}$$

where: $Q^{-1}\left(f\right)$—damping coefficient for the relaxation process as a function of frequency, f—frequency, f_max_—maximum frequency.

**Figure 4:**
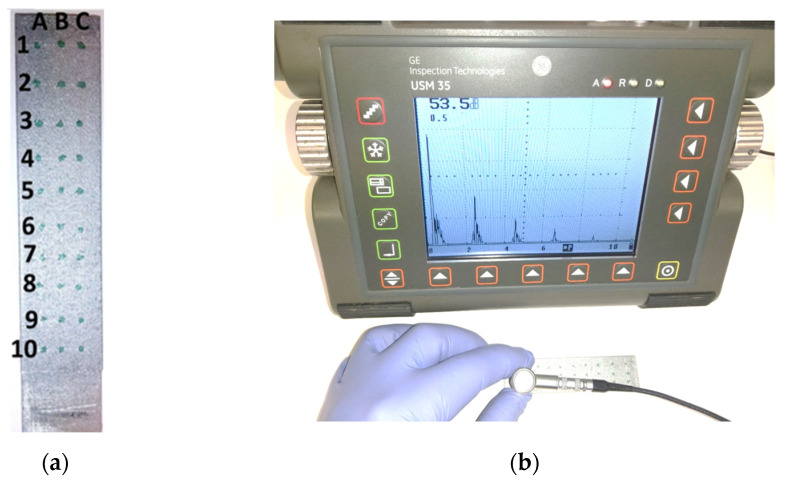
Testing the homogeneity of acoustic parameters of a sample made with additive manufacturing technology; (**a**) view of the sample with the measurement grid, (**b**) view of the test stand.

As the next step, the material made using the additive manufacturing technology was checked for homogeneous acoustic parameters (within one sample). For this purpose, 30 measurement points were determined on two randomly selected samples, in which the pulse systems were recorded on the screen of the ultrasonic flaw detector. The view of the measurement grid used during the research is shown in Figure 4.

Afterwards, it was verified whether the ultrasonic flaw detector settings affected the ultrasonic quality of the adhesive joints. The decibel decreases of the first two pulses from the connection area, described by relationship (4), was adopted as the ultrasonic measure and used during the main research.

(4) $$R=20\cdot \log\left(\frac{H_{I}}{H_{\mathrm{II}}}\right)$$

where: H_I_, H_II_—percentage height of the first and second impulse from the bottom of the tested element.

**Figure 5:**
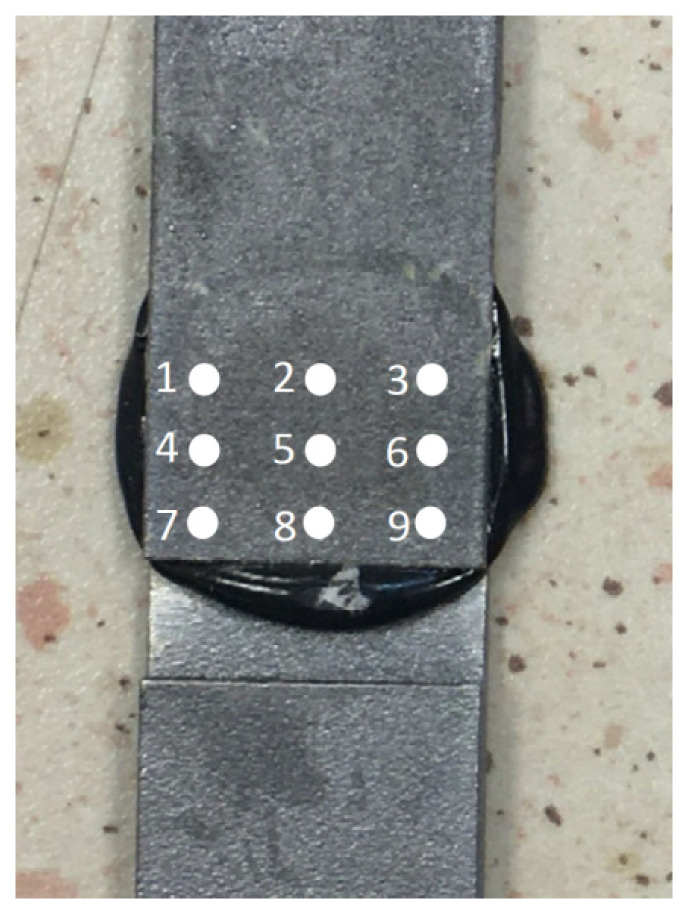
View of the sample with the measuring grid.

After the preparation of the adhesive joints in which adhesive 4 was used, the distribution of the ultrasonic measurement within the entire joint was examined (calculated in accordance with relation (2)). The view of the sample, with the measuring grid marked on it, is shown in Figure 5.

### 2.3. Mechanical Testing of Joints

**Figure 6:**
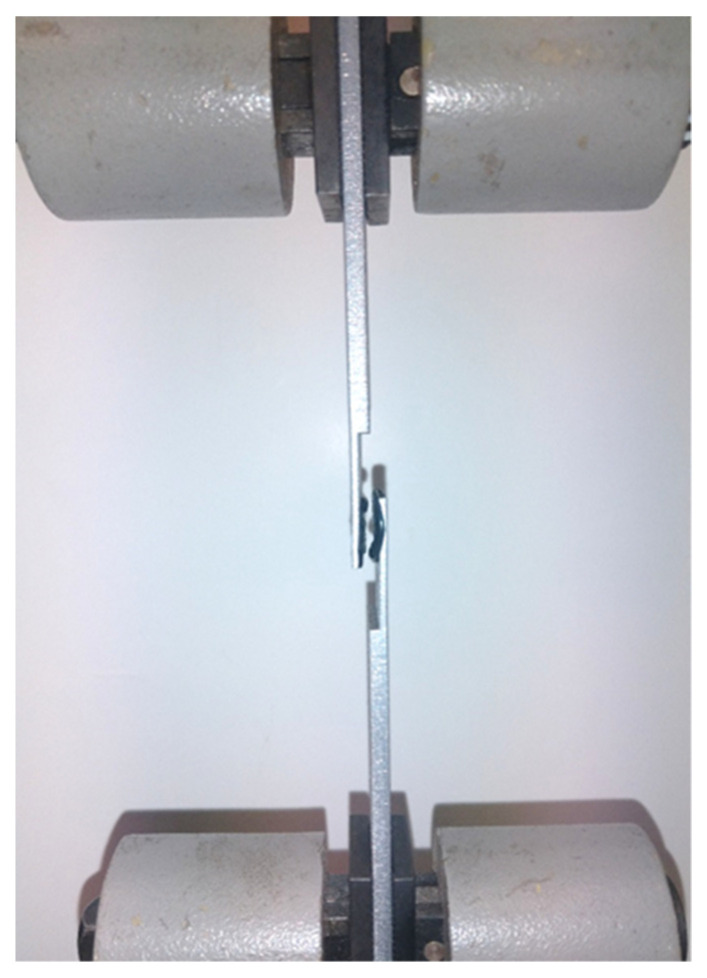
View of the sample after the shear test.

A Cometech B1/E testing machine (Cometech Testing Machines Co., Taichung Taiwan) was used in the tests, together with articulated clamps in which the sample was mounted. The jaw speed was 0.05 mm/s and the force was measured with an accuracy of 1 N. Following that, the maximum shear force obtained during the measurements was related to the surface on which the adhesive had been applied. Thanks to this, the shear stress which damaged the adhesive joint was determined. The view of the sample mounted in the jaws of the testing machine is shown in Figure 6.

### 2.4. Research Plan

**Figure 7:**
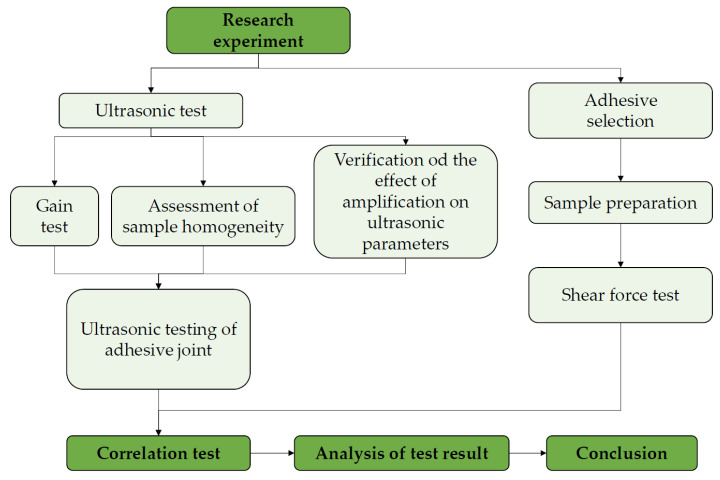
Plan of the research.

The research was divided into two main stages conducted in laboratory conditions. In the first stage, a series of tests were carried out with the use of an ultrasonic wave propagating both in the material itself (sample made with the additive manufacturing technology) and in the adhesive joint. The second stage of the research included destructive tests and a comparison of selected ultrasonic measures with the shear stress. The course of the individual research stages is shown in Figure 7.

## 3. Results

### 3.1. Ultrasonic Testing of Samples’ Results

In the first stage of the ultrasonic tests, the damping measurements of the samples produced with the use of the additive technology were performed. Example results of the height (amplitude) of individual ultrasonic wave pulses assuming a constant height of the first impulse (80% of the height of the ultrasonic flaw detector screen) are summarized in Table 3.

In order to compare the results obtained from the ultrasonic tests of printed samples, a damping value for the sample made of aluminum produced with standard methods was determined (Table 4).

For aluminum samples made with standard methods, two additional pulses were obtained on the ultrasonic flaw detector screen (H_VI_, H_VII_), with the same gain of the ultrasonic wave pulse. It proves a lower attenuation of the material in relation to samples made with the additive technology, where only five return pulses were obtained from the bottom of the sample. These results show that materials made using the 3D printing technology cause a greater attenuation of the ultrasonic wave, due to the internal structure created in the powder remelting process. Moreover, for the sample made with the use of the additive technology, the second pulse from the bottom of the connection was nearly 50% lower than the first one, which proves the high value of the wave attenuation. For samples made with a standard process, the difference between the first and second pulse (height/amplitude) was approximately 20%. The attenuation calculated on the basis of dependence (1) in the elements made with the additive manufacturing technology for the ultrasonic head frequency of 20 MHz was 0.694 dB/mm. The attenuation for aluminum sheet was 0.519 dB/mm (for a frequency of 20 MHz), which is clearly lower than for elements made using the additive technology. This means that conducting ultrasonic tests of elements made with the use of the additive technology is more difficult than conducting tests on elements made with the use of classical methods.

In the next step of ultrasonic testing of elements printed in aluminum, the homogeneity of the acoustic parameters of the samples was measured and selected results are presented in Table 5.

Based on the results presented in Table 5, it can be concluded that the acoustic properties were at a similar level within one sample. In addition, taking into account the test results for all samples, slight (1–2%) differences in the obtained values of the ultrasonic parameters were noticed. Therefore, it can be concluded that the structure of the samples used in the research was practically identical, taking mainly into account the acoustic properties of the AlSiMg0.6-printed material.

**Figure 8:**
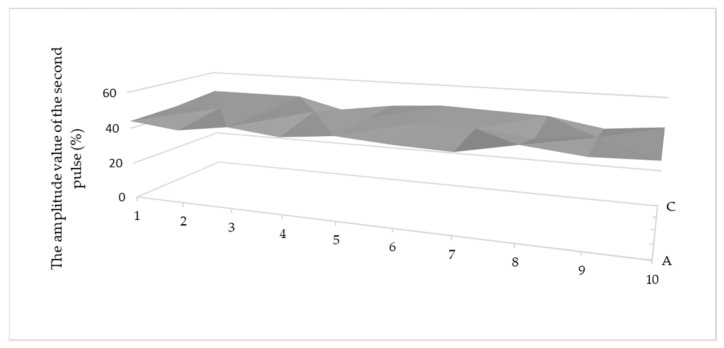
Second pulse amplitude value in % of the ultrasonic flaw detector screen on the whole sample area.

**Figure 9:**
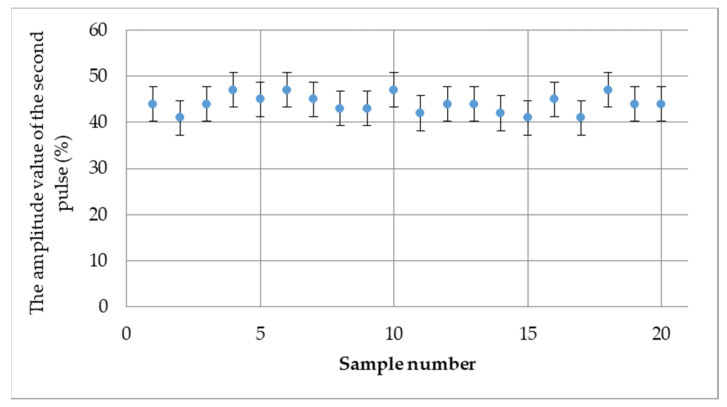
Average amplitude value of second pulse from the sample in % of ultrasonic flaw detector screen.

Figure 8 confirms this above conclusion, showing the amplitude value of the second pulse from the bottom of the connection. The obtained values are at a similar level (based on statistical evaluation), between 40% and 50% of the ultrasonic flaw detector screen height, which is shown in Figure 9.

**Figure 10:**
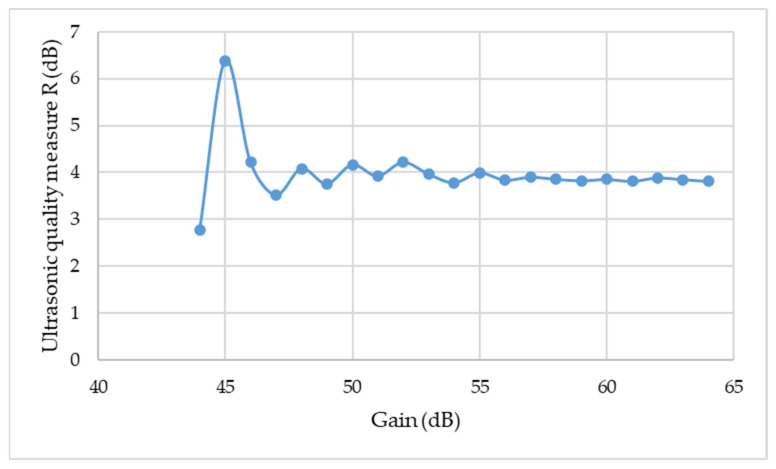
Dependence of the ultrasonic R measure on the amplification of the ultrasonic wave pulse.

The basic parameter of the flaw detector, which affects the pulse height, is the gain of the ultrasonic longitudinal wave, expressed in decibels. Therefore, additional measurements of the height of the first, second and third impulse obtained on the ultrasonic flaw detector screen were made for a gain in the range of 44 dB to 64 dB. At the lowest gain, the height of the first impulse was 11% of the screen height, and the highest one, according to the indications, amounted to 107%. The results of these measurements are summarized in Figure 10. The measured results clearly show that for the tested samples (printed in 3D) an ultrasonic wave impulse amplification above 55 dB of, unchanging results of the measurement of the quality of the sample itself (homogenity) as well as the quality of the adhesive bond were obtained.

### 3.2. Ultrasonic Testing of Adhesive Joints Results

**Figure 11:**
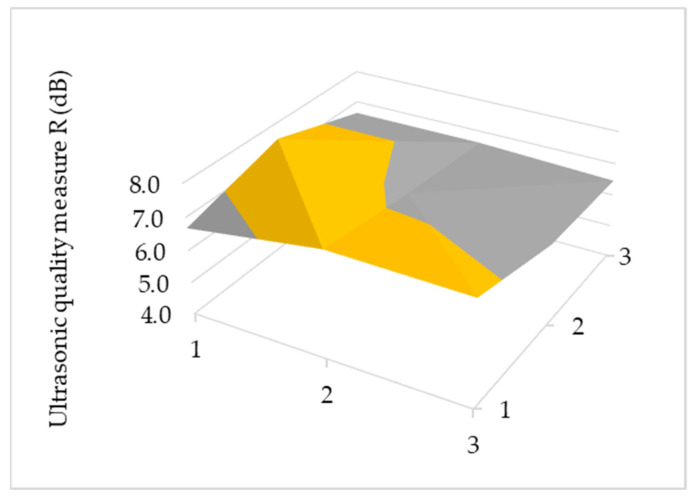
Ultrasonic measure test results.

Ultrasonic tests of adhesive joints were carried out at nine measuring points (Figure 5), determining the ultrasonic measure according to relationship (2). The results of the distribution of this ultrasonic measure for the selected sample are shown in Figure 11.

In the next part of the research, for samples prepared in the same way, the ultrasonic measure of adhesion of the adhesive to the sample substrate was determined. The mean results of all measurements for each of the samples are summarized in Table 6. The results of the ultrasonic measurement for the tested samples ranged from 4.61 to 7.96 dB. This gives a difference of 25%, which may indicate a different adhesion of the adhesive to the surface layer for the 3D printed material. Furthermore, the obtained results should be related to the results of the shear force, which damages the adhesive connection.

### 3.3. Mechanical Testing Results

Before performing the main tests, i.e., shear tests, preliminary tests were carried out to select an adhesive for the main tests. Therefore, the shear stress for all adhesives described in Section 2.1. was determined. For adhesive 1, the average stress was 2.2 MPa, for adhesive 2 it was 12.6 MPa and for adhesive 3 it was 15.7 MPa. The highest value of shear stress which destroyed the adhesive joint was obtained for the epoxy adhesive marked with 4, and it was about 21.7 MPa. On the basis of these tests, it was assumed that adhesive 4 would be used in the main tests. It is a two-component epoxy adhesive which, according to the manufacturer, is characterized by a shear strength for the aluminum–adhesive–aluminum joint of 28–30 MPa (depending on the curing cycle).

By the same token, preliminary tests confirmed that the adhesive joints made on the surfaces which had been only degreased were of the lowest quality. The low quality was due to the oxides formed on the surface. The joints in which the surface was abrasive-blasted (sandblasted) were of much higher quality, while the joints of the highest quality were sanded with the P80 sandpaper (the highest values of stress damaging the adhesive joint). Therefore, in the main tests, this method of sample surface preparation was chosen. Moreover, the method was compatible with the surface preparation possibilities envisaged by the adhesive manufacturer.

**Figure 12:**
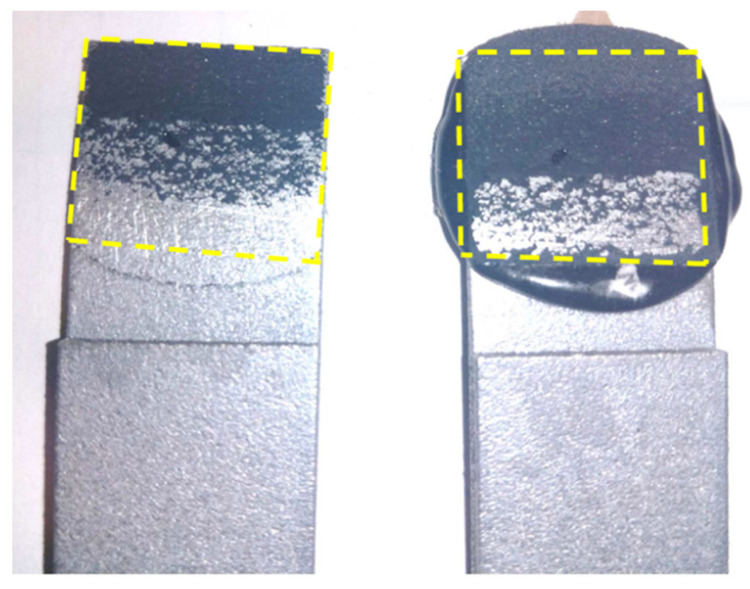
View of the joint after the shear force test.

The results of destructive tests of the joints performed on the testing machine are presented in Table 7. However, the state of one sample after shear test is illustrated in Figure 12—especially the place of the adhesive. In most cases, a cohesive failure was achieved, which means that the forces bonding the adhesive to the substrate were greater than the adhesive bond strength.

## 4. Discussion

**Figure 13:**
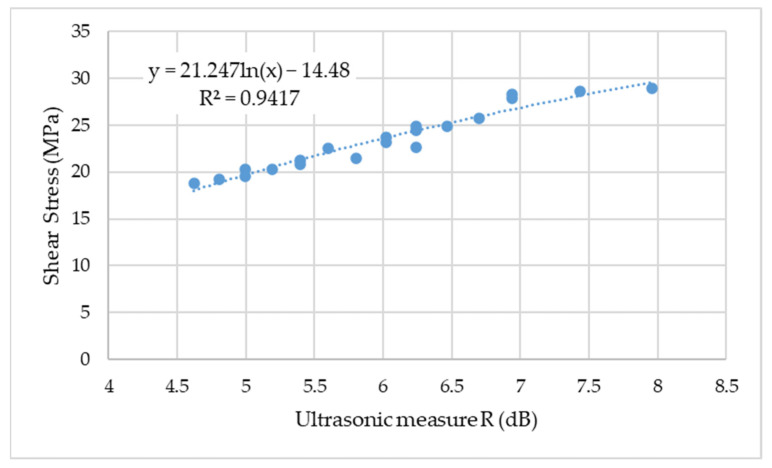
Correlation test results.

The final result of the research was to determine the relationship which combines the ultrasonic quality measure of the adhesive joint with the strength expressed in megapascal determined on the basis of the shear test. This relationship is shown in Figure 13.

The shear stress of the adhesive joints ranged from 18.75 MPa to 28.95 MPa, which corresponds to the ultrasonic measure over a range of 4.61–7.96 dB. The obtained results of shear stress were in accordance with the adhesive data sheet. Nevertheless, it is difficult to unequivocally relate these results to the studies of other authors, due to the characteristics of the samples and the adhesive itself. Sliwa-Wieczorek et al. [39] tested double-lap adhesive connections in destructive shear tests under a quasi-static load at 20 °C and 80 °C. The obtained results of stress destroying the connection were at a similar level as those obtained in the research described by the authors, despite different bonding materials.

In the case of a sample made using the additive technology on which no adhesive was applied, the decibel decrease in the height of the first and second impulse was below 4 dB. However, for the weakest connections, it was 4.6 dB. The coefficient of determination between the decibel decrease in the height of the first and second impulse and the shear stress was high and amounted to 0.94. The obtained relationship can be used to estimate the strength of adhesive connections. However, it should be stated that for the control of other adhesive joints, it is important to take into account the method of surface preparation and the type of adhesive.

An adhesive (glued) bond is a special type of adhesive bond. It has damping and elastic properties which make testing with the use of ultrasound wave difficult. Polyurethane adhesives are characterized by a high damping, and the tested epoxy adhesive is mainly characterized by elastic properties (with lower damping). The properties of the tested materials (glue/adhesive and an element made with additive manufacturing) affect the waveform at the boundary of the connected materials. A part of the wave is reflected, and a part penetrates through the adhesive bond. This phenomenon can be described by the energy reflection coefficient R_E_—Equation (5). It is the ratio of the energy of the reflected wave to the energy of the incident wave. The energy carried by the ultrasonic wave is proportional to the square of the amplitude of the sound pressure, the energy reflection coefficient is equal to the square of the pressure coefficient and can take the form of the expression:

(5) $$R_{E}=\frac{E_{1}}{E_{0}}=\frac{{\left(z_{2}-z_{1}\right)}^{2}}{{\left(z_{2}+z_{1}\right)}^{2}}$$

where: E_0_, E_1_—energy carried by the incident and reflected wave, respectively; z_1_, z_2_—acoustic impedance of the first and second medium, respectively.

The actual value of the reflectance is influenced by many factors, e.g., the preparation of the surface for bonding, the setting conditions, the bonding and cross-linking temperature. For this reason, the analytically determined reflectance value is only a guide for the ultrasonic quality evaluation of the adhesive joint. The higher the quality of the adhesive bond is, the greater part of the ultrasonic wave energy will be attenuated in the adhesive. A smaller part of the ultrasonic wave energy will be reflected from the adhesive bond and will return to the ultrasonic transducer, generating pulses on the flaw detector screen, hence the nature of the adopted measure and its marked increase along with the increase in destructive stresses.

## 5. Conclusions

As part of this work, a research experiment was planned and carried out. The damping of the ultrasonic wave in the samples made with an additive technology was determined. The influence of the ultrasonic wave pulse amplification on the results of the selected ultrasonic measure was also assessed. In the next part, tests on the destructive quality of the adhesive joint were carried out and the obtained results were compared with the nondestructive measurement of the quality of the joint. The most important conclusions from the conducted research are as follows:

- The shear stress of the tested adhesive joints ranged from 18 to almost 29 MPa;
- The lowest decibel drop between the first two pulses was 4.6 and the highest one was almost 8 dB;
- The coefficient of determination between the ultrasonic measure and the mechanical measure of the tested connections was 0.94.

The application of additive manufacturing technologies creates new opportunities of manufacturing complex geometries without increasing costs. The additional benefits of using additive manufacturing technologies could be achieved thanks to the implementation of topology optimization methods such as the biomimetic structural optimization approach [40].

Further work should include research on the propagation of ultrasonic waves in adhesive joints for adhesives with different acoustic and mechanical properties, e.g., cyanoacrylates. Such connections may have a different course of pulses from the connection area and may allow the use of other ultrasonic measures. Subsequent research will enable extending the database of the results of adhesive joints’ assessment using the ultrasonic method, which will contribute to the development of a system for assessing the strength of an adhesive joint without damaging it.

## Figures and Tables

**Table 1:** Chemiacal composition (mass fractions in %).

Al	Cu	Fe	Mg	Nb + Ta	Mn	Si	Ti	N	Zn	Each Other	Total Other
Balance	0.05	0.19	0.45–0.70	/	0.10	6.50–7.50	0.25	/	0.07	0.03	0.10

**Table 2:** Selected properties of adhesives used in research.

Property	Adhesive 1	Adhesive 2	Adhesive 3	Adhesive 4
Setting time (20 °C)	4 h	2 h	1 h	4–6 h
Cure time (20 °C)	48 h	24 h	24 h	7 days
Shear strength	4.5 MPa	3.1 MPa	20–24 MPa	30.2 MPa
Shore hardness	50 ShA	77 ShA	73 ShD	81 ShD
Adhesive type	Polyurethane prepolymer	Hybrid adhesive	Methacrylate adhesive	Epoxy adhesive

**Table 3:** The results of ultrasonic measurements used to determine the attenuation in samples produced with the additive technology (the complete set of results are available in the Appendix A in Table A1).

H_I_ (%)	H_II_ (%)	H_III_ (%)	H_IV_ (%)	H_V_ (%)
Mean value	45	24	12	6
Standard deviation	2.08	1.53	1.22	0.90

**Table 4:** The results of ultrasonic measurements used to determine the attenuation in samples produced using standard technology (the complete set of results are available in the Appendix A in Table A2).

H_I_ (%)	H_II_ (%)	H_III_ (%)	H_IV_ (%)	H_V_ (%)	H_VI_ (%)	H_VII_ (%)
Mean value	63	48	34	25	17	12
Standard deviation	1.89	3.39	3.01	2.68	2.63	2.10

**Table 5:** Results of measurements of the homogeneity of selected acoustic properties of samples made using additive manufacturing technology (the complete set of results are available in the Appendix A in Table A3).

Sample 1				Sample 2		
Mean value	45.07	12.83	7.43	44.83	13.00	7.23
Standard deviation	2.21	2.11	1.20	2.42	2.34	1.02

**Table 6:** Average results of ultrasonic measure for all samples (the complete set of results are available in the Appendix A in Table A4).

	Ultrasonic Measure (dB)
Mean value	6
Standard deviation	0.88

**Table 7:** Results of shear stress for all samples (the complete set of results are available in the Appendix A in Table A5).

	Shear Stress (Mpa)
Mean value	23.363
Standard deviation	3.18

## Data Availability

The data presented in this study are available on request from the corresponding author.

## Appendix A

**Table A1:** The results of ultrasonic measurements used to determine the attenuation in samples produced with the additive technology.

H_I_ (%)	H_II_ (%)	H_III_ (%)	H_IV_ (%)	H_V_ (%)
80	45	23	13	6
80	46	24	11	7
80	45	22	12	5
80	44	23	11	6
80	45	22	11	4
80	45	24	12	6
80	48	26	12	6
80	44	21	9	4
80	43	24	11	6
80	49	25	13	7
80	43	22	12	5
80	49	26	13	6
80	45	22	11	5
80	45	23	11	6
80	43	23	13	7
80	42	22	11	5
80	45	23	11	6
80	45	24	12	7
80	43	21	11	6
80	48	28	16	8
80	50	26	14	8
80	46	24	13	7
80	46	24	13	6
80	43	23	12	7
80	45	22	12	6
80	47	24	12	6
80	47	22	11	5
80	43	22	11	7
80	44	24	13	6
80	44	24	12	7

**Table A2:** The results of ultrasonic measurements used to determine the attenuation in samples produced using standard technology.

H_I_ (%)	H_II_ (%)	H_III_ (%)	H_IV_ (%)	H_V_ (%)	H_VI_ (%)	H_VII_ (%)
80	63	46	29	20	12	8
80	65	48	33	22	11	9
80	63	50	38	25	18	15
80	60	46	34	24	16	10
80	60	44	33	22	18	11
80	63	60	43	34	24	17
80	63	45	35	26	20	14
80	61	48	36	25	19	13
80	63	49	36	27	18	14
80	63	45	37	26	19	13
80	69	52	38	28	19	14
80	64	46	36	26	19	13
80	60	42	29	19	11	8
80	62	45	33	23	16	10
80	63	47	34	25	18	12
80	65	49	34	25	18	13
80	63	44	34	26	17	12
80	63	43	31	28	16	10
80	64	47	34	25	18	14
80	66	51	36	27	18	13
80	63	50	38	25	18	15
80	63	49	36	27	18	14
80	66	49	34	24	19	13
80	64	48	33	24	17	13
80	62	49	30	25	14	14
80	63	49	30	24	16	14
80	62	51	29	23	15	14
80	61	43	33	24	17	11
80	65	47	31	22	13	11
80	62	45	34	24	17	10

**Table A3:** Results of measurements of the homogeneity of selected acoustic properties of samples made using the additive manufacturing technology.

Sample 1					Sample 2				
No	H_I_ (%)	H_II_ (%)	H_III_ (%)	H_IV_ (%)	No	H_I_ (%)	H_II_ (%)	H_III_ (%)	H_IV_ (%)
1	80	44	15	9	1	80	45	11	9
2	80	41	13	7	2	80	42	12	8
3	80	45	13	6	3	80	45	16	7
4	80	42	15	9	4	80	44	14	7
5	80	45	11	9	5	80	42	12	6
6	80	43	13	8	6	80	43	12	9
7	80	42	15	8	7	80	42	16	6
8	80	48	13	7	8	80	43	14	9
9	80	45	9	6	9	80	47	12	7
10	80	46	15	6	10	80	48	10	9
11	80	45	13	8	11	80	43	15	7
12	80	46	15	6	12	80	48	9	6
13	80	46	11	8	13	80	41	10	7
14	80	48	13	8	14	80	47	15	9
15	80	46	14	6	15	80	43	14	7
16	80	46	14	9	16	80	49	10	6
17	80	45	16	7	17	80	46	12	8
18	80	42	16	7	18	80	43	15	7
19	80	45	12	6	19	80	49	15	6
20	80	45	9	9	20	80	47	16	8
21	80	48	14	6	21	80	47	16	8
22	80	48	12	7	22	80	47	16	7
23	80	48	10	9	23	80	42	9	7
24	80	41	9	6	24	80	43	11	8
25	80	46	13	6	25	80	42	14	7
26	80	48	14	9	26	80	49	13	7
27	80	47	11	8	27	80	46	9	7
28	80	46	16	6	28	80	44	14	6
29	80	41	10	8	29	80	44	12	6
30	80	44	11	9	30	80	44	16	6

**Table A4:** Average results of ultrasonic measure for all samples.

Sample Number	Ultrasonic Measure (dB)
1	6.021
2	4.998
3	6.936
4	5.806
5	7.959
6	5.392
7	6.466
8	5.193
9	4.807
10	5.597
11	6.241
12	6.936
13	6.241
14	6.241
15	4.620
16	5.392
17	7.432
18	6.021
19	6.698
20	4.998

**Table A5:** Results of shear stress for all samples.

Sample Number	Shear Stress (Mpa)
1	23.212
2	19.580
3	27.900
4	21.480
5	28.950
6	21.240
7	24.915
8	20.250
9	19.250
10	22.490
11	22.650
12	28.272
13	24.495
14	24.850
15	18.750
16	20.812
17	28.565
18	23.687
19	25.680
20	20.240
